# Priority-setting, the Indian way

**DOI:** 10.7189/jogh.08.020311

**Published:** 2018-12

**Authors:** Neethi V Rao, Laura Downey, Nishant Jain, Rama Baru, Francoise Cluzeau

**Affiliations:** 1 Imperial College, London, UK; 2 International Decision Support Initiative, London, UK; 3 Deutsche Gesellschaft für Internationale Zusammenarbeit, Delhi, India; 4 Jawaharlal Nehru University, Delhi, India

The Twelfth Five Year Plan of India sets out the ambitious agenda of improving the availability, quality and affordability of health services to initiate the move towards Universal Health Coverage (UHC) [1]. With this in mind, the government of India has announced its *‘Ayushman Bharat’* programme to be rolled out with a National Health Protection Mission (PMJAY) targeting 100 million poor families for insurance coverage of up to INR 500 000 per annum [2]. The PMJAY is to be supplemented with directed efforts to strengthen primary care and medical education across the country [2]. Operationalising this ambitious programme will require significant investment of public resources into healthcare. The National Health Policy (2017) has explicitly committed to increase India’s government health spending to 2.5% of its GDP by 2025. However, India’s public health spending continues to be close to 1.2% of GDP, demonstrating the limited fiscal space available for health [3]. Operationalising UHC will require optimal utilisation of existing resources to ensure that the greatest amount of health is bought for every rupee spent.

India has a pluralistic health system with over 70% of the care delivered through the private sector [4]. The private sector is highly diverse and ranges from large corporate-style multi-speciality hospitals, to not-for-profit charitable institutions, to single clinics manned by private doctors. The standard of care, whether offered in the public or private sector, is highly heterogeneous with world-class hospitals co-existing with semi-trained practitioners. Given this complexity, governments in India at the state and central levels have dual responsibilities. On the one hand, they need to ensure that public resources are budgeted and allocated efficiently to have the maximum possible impact on health coverage. On the other, public policy needs to nudge and induce better healthcare provisioning by diverse providers. Fulfilling these responsibilities requires systematic policy guidance that incorporates scientific evidence and sound governance processes to identify good value and good quality health interventions.

The government has recognised this need and embraced health technology assessment (HTA) as a systematic policy tool for priority-setting. Health technology assessment (HTA) involves comparative assessments of health interventions, incorporating evidence related to clinical and cost effectiveness, safety, social, political and ethical considerations associated with given health interventions to help identify the best alternative. HTA tells us whether current intervention strategies represent an efficient use of scarce resources, and which of the potential interventions that may be implemented should be prioritised. The Department of Health Research (DHR) under the Ministry of Health and Family Welfare (MoHFW), Government of India, has recently set up the country’s first HTA agency , the HTAIn [5] that will commission multi-disciplinary studies by trained research groups across the country [6]. Interest and capacity of research institutions across the country was systematically probed and researchers are being actively encouraged to develop capacity, critically engage and contribute to the technical processes of priority-setting in India.

The newly formed body will respond to the needs of government decision-makers at state and central levels by providing evidence-based policy guidance [5]. As the use of HTA develops in the country, it can help improve policy design in line with defined policy objectives of ensuring value for money of public resources. Increased transparency, effective engagement with stakeholders, and careful management of potential conflicts of interest, will be essential components in establishing the legitimacy of the HTAIn process.

Health policy-making in India is segmented both horizontally and vertically across many different agencies and departments. Constitutionally, health is defined as a subject under the jurisdiction of state governments. However, the central government also plays a key role in making resources available, in design and technical support. Additionally, ministries such as Defence, Labour and Railways may run their own hospitals and health facilities for providing services to their respective constituencies. Further, closely allied functions such as pricing of drugs and devices are governed by ministries other than the Health Ministry at the central level. Thus, there are multiple potential users for HTA in India at state and central levels, including health departments, insurers, procurement agencies, hospital administrators and providers. Each of these policy-making agencies represent potential users of HTA evidence to improve priority-setting within their respective functional contexts. There are myriad ways in which HTA evidence can be used to strengthen the priority-setting process at each level of the decision-making space in Indian health system. With the nascent establishment of HTA as a legitimate component of the priority-setting process in India, there is a pressing need to ensure the efficient and effective deployment of HTA evidence into the policy process to maximise uptake and value.

In this paper, we outline the many different uses of HTA corresponding to various categories of policy-makers within the current organisational structure in India. We do not seek to analyse the methodological aspects associated with actually conducting health technology assessments in the Indian context. Instead, by detailing the potential applications of HTA in defined policy contexts, we explore the value of evidence-based priority setting in achieving strategic goals within the complex health system of India.

## TYPES OF DECISION MAKERS AND THE USES OF HTA IN INDIA

**Figure 1:**
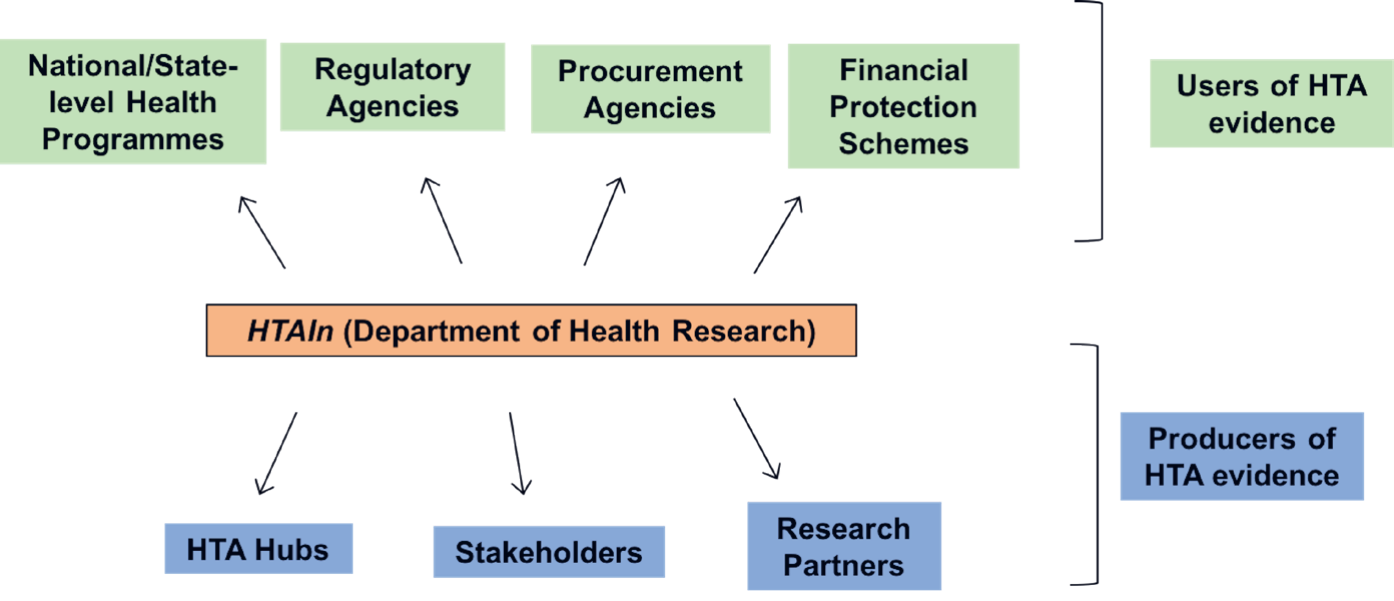
Stakeholders for HTA in India.

Countries across the world have various organizational mechanisms for HTA use within their respective health systems. Countries such as the United Kingdom and Thailand have national HTA agencies that support policy-making for the entire country [7,8]. Others such as Italy and Canada have experimented with HTA use at national and sub-national jurisdictions with varied degrees of impact [9,10]. India as a federal system with shared responsibilities for healthcare decision-making, multiple systems of medicines and a large private sector presents both challenges and opportunities for the creation of a unique model of HTA use. HTAIn is set up by the national government to serve as the secretariat for the HTA programme in India. **Figure 1** represents the organizational model that HTAIn is applying for early adoption and implementation of HTA in the country. HTAIn will commission, generate, quality assure and ratify HTA evidence; functioning as a focal point between users and producers of evidence.

There are a number of ways that HTA may inform key decision making for health in India both in the public and the private sector. In this paper, we focus on the uses of HTA for governmental policy making. However, it is important to note that private sector actors have key roles to play as both users and producers of HTA evidence. Data generated through insurance companies, hospitals and providers are essential inputs to improving the quality of assessments. Providers in the public and private sector need to embrace HTA evidence and incorporate it into their practice.

**Table 1** outlines each of the different categories of users for HTA evidence with examples of user functions. As there are multiple organizational structures performing similar functions within the complex Indian system (especially at state government levels), we do not comprehensively list each potential user, and have instead chosen illustrative examples that can be more generally applied to similar institutions.

**Table 1:** Examples of specific uses of HTA for different government health authorities

Category	Examples	Uses of HTA
Targeted health programmes	• National Health Mission	• Rationalise components and Identify the most cost-effective package of interventions under each programme to maximize health gain
	• State Health Programmes	• Assist in procurement by identifying most cost-effective drugs & devices
		• Identify required budget allocations to achieve goals of each programme
Financial Protection programmes	• National Health Protection Mission	As above, and: • Develop threshold for reimbursement using health benefits obtained per rupee spent.
	• State Schemes	• Developing cost-effective standard treatment guidelines to improve quality of care
		• Assist in quality regulation of empanelled hospitals by informing quality metrics for reimbursement
		• Informing pay for performance standards of practice
Procurement Agencies	• Supplier Corporations	• Improve procurement by identifying cost-effective list of products and services
	• State Health Departments	• Assist in strategic purchasing by identifying cost-effective costs and prices
		• Remove duplications or waste and rationalise stock based on volume of usage
Regulatory Agencies	• Clinical Establishments Act	• Assist in development of contextually relevant quality metrics for service provision
	• National Pharmaceutical Pricing Authority (NPPA)	• Rationalise list of medicines in the National List of Essential Medicines (NLEM)
		• Assist in deriving cost-effective pricing for drugs

## HOW HTA CAN SUPPORT GOVERNMENTAL STRATEGIC OBJECTIVES FOR HEALTH SYSTEM STRENGTHENING IN INDIA

Improving the way in which health services are financed, procured, delivered, and governed, have all been identified as priority areas for the government [1,11,12]. When utilised well, HTA can have a role to play in each of these broad agendas. Here we outline how HTA supports these strategic objectives towards strengthening the system as a whole.

### 1. Facilitating strategic purchasing of services from the private sector

Strategic purchasing requires priority-setting decisions around what to purchase, from whom and at what price. The private sector is the dominant care provider in India and strategic purchasing of services from the private sector is one of the key strategies that the Government is adopting to achieve UHC in India [2,11,13]. The National Health Protection Mission (PMJAY), for example will empanel private hospitals to deliver health benefits that will be reimbursed up to a limit of INR 500,000 (7700 USD) [2]. HTA can provide a valuable input into the design of the PMJAY benefits package by prioritising high value interventions to maximise outcomes of health and financial risk protection [14]. State government health insurance schemes such as the *Swasthya Sathi* scheme in West Bengal or *Bhamashah* scheme in Rajasthan that are already operational may also use HTA to determine the appropriate price for reimbursement by identifying the comparative value of alternative health interventions. While HTA alone cannot overcome limitations of poor governance or lack of regulatory oversight on private provision of healthcare, it provides rational grounds for policymakers to negotiate appropriate terms for strategic purchasing.

### 2. Incorporating value-based pricing for drugs and devices

HTA evidence can be used to support value-based pricing by incorporating cost-effectiveness of drugs and medical devices in the price-setting process [15]. Regulatory approval for drugs in India is primarily based on the three criteria of quality, safety and efficacy. In addition, India imposes price control on a select set of drugs and devices through the National Pharmaceutical Pricing Authority (NPPA) [16]. The price control policy along with the patent regime have contributed to some of the lowest prices for drugs in India [17]. Price control policies for essential drugs are an important component of ensuring affordability in a country that remains largely poor with over 70% of healthcare costs being paid out-of-pocket. In addition to the domestic market, India as the ‘pharmacy of the developing world’ also affects the availability of affordable medicines globally. However, the current pricing negotiations are often criticized by stakeholders for leading to perverse incentives [18,19]. Value-based pricing incentivizes innovation and drug development instead of being barriers and as such is beneficial for all parties [15]. However, determining the value of drugs and devices maybe challenging given the widespread misuse of medication in India and the lack of data on treatments and outcomes. Development of a systematic evidence-based priority-setting architecture will require development of a data infrastructure that enables tracking of pharmaceutical use and healthcare delivery and in turn, checks irrational drug use and malpractice. Combined with stronger regulation and increased public investments, value-based pricing supported by HTA can help improve access to medicines.

### 3. Improving quality of care

HTA can help in the improvement of quality of care in two ways. First, HTA can inform the development of cost-effective standardised care pathways [20] (reference s21 in the list of references in **Online Supplementary Document(Online Supplementary Document)**). Second, HTA can be used to inform reimbursement criteria for purchasing clinical services, thereby improving care by requiring HTA-informed quality standards to be met (s22). The government of India is exploring policy instruments to incentivise accreditation and standardised care pathways to institutionalise health service quality (s23,s24). HTA can assist in the process of development of contextually relevant clinical guidelines that maybe used for accreditation or other regulatory instruments such as payment for performance. The use of HTA ensures that standards are evidence-based and have the buy-in of appropriate stakeholders, facilitating compliance. This is especially crucial in a diverse health system such as in India with multiple systems of medicine including Ayurveda, Unani and Homeopathy. When adequately enforced, these standards increase the consistency and reliability of healthcare.

### 4. Regulation of healthcare provision

The use of HTA strengthens the regulatory power of government agencies by providing levers to regulate the price, quality and distribution of health services across the system (s25). The pharmaceutical, medical technology, diagnostics and hospital industries together wield a powerful influence on public policy and practice in India. Use of evidence in decision-making tempers this influence by justifying regulatory actions in the interest of larger policy goals. HTA can assist in results-based financing for health interventions in the public or private sector based on their ‘value’ or utility in the health system. Since ‘value’ of any health intervention is only relevant within the context of the care pathway and the target population, HTA can help design appropriate outcome and quality indicators to ensure payment is adjusted to performance.

Established HTA agencies across the world are increasingly building policy linkages between HTA and regulation of healthcare quality (s26). India has the opportunity to learn from those experiences and establish those pathways at an early stage.

**Figure:**
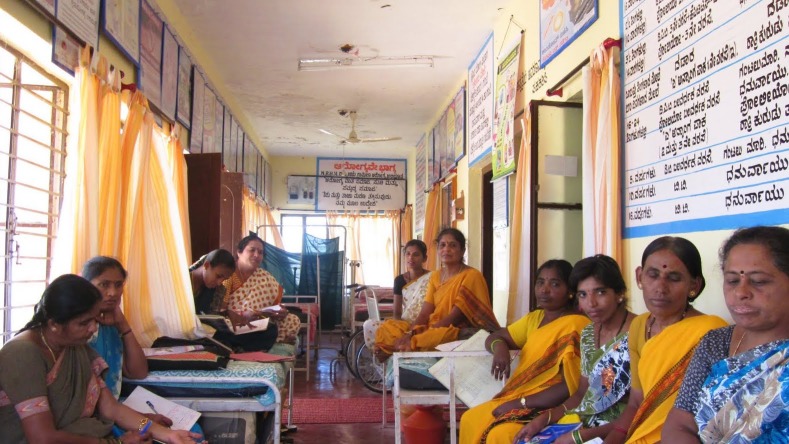
Photo: from the Institute of Public Health, Bengaluru, India (used with permission)

### 5. Achieving policy convergence and cooperation

HTA helps measure how efficient a given health intervention is compared to all reasonable alternatives. In other words, it helps identify which of the available alternatives maximises health outcomes. Conversely, it also uncovers health interventions whose costs outweigh the benefits derived from them. HTA evidence on efficiency of government health programmes can be used to help rationalise interventions at state or national levels. Different health schemes/programmes in India sometimes implement similar or overlapping interventions. For example, two separate government ministries in India use similar interventions to provide affordable access to medicines. The *Jan Aushadi* campaign launched by the Department of Pharmaceuticals under the Ministry of Chemicals & Fertilizers involves the setting up of retail pharmacies for generic medicines at affordable prices. More recently the Ministry of Health have now begun setting up Affordable Medicines and Reliable Implants for Treatment (AMRIT) pharmacies to sell subsidised medicines. While each programme is laudable in its own right, an evidence-based analysis of the effectiveness and efficiency of low-cost pharmacies in the country could improve the policy design and facilitate complementarity and convergence across government schemes/programmes. HTA can also facilitate better health policy cooperation between state and central governments by increasing the efficiency of resource allocations and helping identify areas of complementarity. This is increasingly important in light of the increased fiscal devolution from the centre to the states.

### 6. Incorporating concerns of equity and social justice in health policy decisions

HTA provides a mechanism to systematically incorporate evidence on health inequities, ethics and implementation challenges into priority-setting that is best suited to the relevant population context (s27). The normative judgements and priorities of the government underpin every one of the choices made in the assessment, offering a policy reflection of societal values e.g. assessments may disaggregate costs and benefits of the same health intervention for different population groups based on equity concerns or use outcome measures beyond healthcare that incorporate societal objectives in the determination of priorities for resource allocation. Additionally, the institutional use of HTA in public policymaking can serve as a long-term mechanism to increase public participation and build accountability among citizens, policymakers and health service providers (s28).

India is a highly diverse country with significant inequality along the lines of income, gender, caste and geography etc (s29,s30). Additional interventions maybe needed to achieve similar objectives in tribal areas compared to non-tribal ones or diseases may manifest differently in men versus women. HTAIn thus explicitly includes the objectives of improving financial protection and minimising health inequality in addition to maximising health in decision-making [5]. More broadly, the HTA process provides a unique platform for specific and ongoing policy consideration of dimensions of social justice, where no other such mechanism exists.

## CHALLENGES OF USING HTA IN INDIA

Sustained use of evidence-based priority-setting has transformational potential for India’s health systems by increasing the legitimacy, power and accountability of government policy. However, HTA alone cannot by itself provide a panacea for all the deficiencies within the Indian health system. There remain several challenges for institutionalizing the use of priority-setting tools like HTA in health policy making in India.

The predominance of the private sector in the Indian health system combined with distributed decision-making moderates the impact of governmental agencies such as HTAIn. In a mixed health system such as India’s where over 70% of the care is provided by the private sector, [4,13] all decisions taken by the government will inevitably impact private provision of care. As such, all uses of HTA will impinge on regulation and incentivisation in the healthcare market, whether public or private. This brings challenges associated with lobbying and the inevitable push-back on decisions contrary to the interests of organized interest groups, particularly in the private sector. Additionally, India suffers from issues related to neglect of primary care, medical malpractice, shortage of trained health professionals and poor implementation of regulations [13]. Strengthening of the public sector, the government’s regulatory will and building a healthy public-private working relationship is essential to ensure the long-term relevance and effectiveness of HTA-based decision-making. This requires a strong commitment to transparency and public accountability, accompanied by legislative support to protect against conflicts of interest.

HTA has significant data and technical requirements that requires the rapid development of a robust data infrastructure that is currently absent in many low, middle income countries including India (s21,s31). This is particularly true for public health programmes whose costs and benefits would have to be followed over longer time periods relative to interventions such as drugs or vaccines. It can also be difficult to quantify externalities associated with government initiatives, which impact on health.

There is also limited human resource capabilities in health economics, mathematical modelling and evidence synthesis, requiring considerable investment in skill-building (s32). The government has already recognized this and has actively focused on consolidating available data, commissioning more studies to support growing data requirements as well as capacity building of human resources to conduct and interpret HTA studies. Concerted efforts are also ongoing for the adoption of electronic record keeping of health data that can be incorporated in analyses such as HTA.

Considerations of social justice and health inequities may also be especially challenging given the exceptional diversity of India. Methodologies to systematically consider social justice issues are still evolving (s33) and there is very little international experience with priority-setting that considers factors beyond income or gender inequalities. The intersectionality of issues associated with caste, gender, income and geography are likely to lead to highly contested decision rules (s30). The normative rules can only evolve over a period of time through an internal process of debate among all stakeholders.

## CONCLUSION

The Indian health system presents a unique case of a diverse population with distributed policy-making authority. Tools of priority-setting such as HTA need to be adapted to this context, and could, through iterative practice and evolution, raise both the quality and cost-effectiveness of health care provision. In this paper, we have outlined how decision makers in various government agencies in India may apply HTA to maximise the value of their investments. We specified the particular policy objectives of the Indian health system that institutionalized use of HTA may help achieve, while identifying the overarching challenges to systematic evidence-based priority-setting.

It is important to note that beyond the instrumental uses of HTA, as described in this paper, the iterative use of policy-oriented research has conceptual and symbolic significance for stakeholders across the spectrum. Institutions like HTAIn make the criteria for decision-making explicit and allow systematic, periodic stakeholder input into policy-making thus increasing transparency and public accountability. This also increases the legitimacy of the policy-making process, providing faith to the citizenry that its values and interests are represented in the health system. Institutional use of evidence in public policymaking can help improve overall health system performance and put India on the trajectory to achieving universal health coverage.
