# Supplementary material for: Priority-setting, the Indian way
Source: J Glob Health. 2018 Nov 15;8(2):020311. doi: 10.7189/jogh.08.020311 (PMC6237104; doi:10.7189/jogh.08.020311)
Supplement: Online Supplementary Document [file jogh-08-020311-s001.pdf]

Online Supplementary Document
Rao et al. Priority-setting, the Indian way

J Glob Health 2018;8:020311

Supplementary References:

[s21] Kumar M, Ebrahim S, Taylor FC, et al. Health technology assessment in India: the potential for improved healthcare decision-making. Natl. Med. J. India. 2014;27:159–163.

[s22] Drummond M, Jonsson B, Rutten F. The role of economic evaluation in the pricing and reimbursement of medicines. Health Policy (New. York). 1997;40.

[s23] NITI Aayog. Healthy States, Progressive India: Report on the Ranks of States and Union Territories. 2018.

[s24] National Health Mission (Government of India). Standard Treatment Guidelines [Internet]. [cited 2018 Mar 20]. Available from: http://www.nhm.gov.in/nrhm-instate/520-standard-treatment-guidelines.html.

[s25] Banta D. The development of health technology assessment. Health Policy (New. York). 2018;63:121–132.

[s26] Quality and accreditation in health care services: A global review. World Health Organization, Geneva; 2003.

[s27] International Network of Agencies for Health Technology Assessment (INAHTA). Ethical Issues in HTA [Internet]. [cited 2018 Mar 21]. Available from: http://www.inahta.org/hta-tools-resources/ethics/.

[s28] WHO Consultative Group on Equity and Universal Health Coverage. Making fair choices on the path to universal health coverage: Final report of the WHO Consultative Group on Equity and Universal Health Coverage. Health Econ. Policy. Law. 2014;1–7.

[s29] Balarajan Y, Selvaraj S, Subramanian S V. Health care and equity in India. Lancet. 2011;377:505–515.

[s30] Ravindran S, Gaitonde R, Evidence R. Health Inequities in India. 2018.

[s31] Downey L, Rao N, Guinness L, et al. Identification of publicly available data sources to inform the conduct of Health Technology Assessment in India [version 1; referees: 1 approved]. F1000Research. 2018;7.

[s32] Prinja S, Chauhan AS, Angell B, et al. A Systematic Review of the State of Economic Evaluation for Health Care in India. Appl. Health Econ. Health Policy. 2015;13:595–613.

[s33] Dukhanin V, Searle A, Zwerling A, et al. Integrating social justice concerns into economic evaluation for healthcare and public health: A systematic review. Soc. Sci. Med. 2018;198:27–35.
